# Overexpression of Purinergic P2X4 Receptors in Hippocampus Rescues Memory Impairment in Rats with Type 2 Diabetes

**DOI:** 10.1007/s12264-020-00478-7

**Published:** 2020-03-20

**Authors:** Ping-An Zhang, Qian Sun, Yong-Chang Li, Rui-Xia Weng, Rui Wu, Hong-Hong Zhang, Guang-Yin Xu

**Affiliations:** 1 Jiangsu Key Laboratory of Neuropsychiatric Diseases and Institute of Neuroscience, Soochow University, Suzhou, 215123 China; 2 Center for Translational Medicine, The Affiliated Zhangjiagang Hospital of Soochow University, Suzhou, 215600 China; 3 The Second Affiliated Hospital of Soochow University, Suzhou, 215004 China

**Keywords:** Microglia, P2X4 receptors, DNA damage, Type 2 diabetes mellitus, Memory impairment

## Abstract

Purinergic receptors have been reported to be involved in brain disorders. In this study, we explored their roles and mechanisms underlying the memory impairment in rats with type 2 diabetes mellitus (T2DM). T2DM rats exhibited a worse performance in the T-maze and Morris water maze (MWM) than controls. Microglia positive for P2X purinoceptor 4 (P2X4R) in the hippocampus were reduced and activated microglia were increased in T2DM rats. Long Amplicon PCR (LA-PCR) showed that DNA amplification of the *p2x4r* gene in the hippocampus was lower in T2DM rats. Minocycline significantly reduced the number of activated microglia and the mean distance traveled by T2DM rats in the MWM. Most importantly, P2X4R overexpression suppressed the activated microglia and rescued the memory impairment of T2DM rats. Overall, T2DM led to excessive activation of microglia in the hippocampus, partly through the DNA damage-mediated downregulation of P2X4Rs, thus contributing to memory impairment.

**Electronic supplementary material:**

The online version of this article (10.1007/s12264-020-00478-7) contains supplementary material, which is available to authorized users.

## Introduction

Diabetes mellitus (DM) is one of the most common metabolic disorders and its prevalence in adults has been increasing rapidly in the last decade [1]. Its prevalence is predicted to increase to 642 million people (1 in 10 adults) by 2040 [2]. The rapid transition in lifestyle to urbanization has been accompanied by increases in the risk factors for non-communicable diseases like type 2 DM (T2DM) [3]. DM has been associated with an increased risk of developing Alzheimer disease (AD) and can affect cognitive systems [4, 5]. Specifically, people with T2DM often (but not invariably) do poorly on measures of learning and memory, whereas deficits in these domains are rarely seen in people with type 1 diabetes [6]. Despite the increased risk of AD in patients with T2DM [7], emerging evidence shows that such patients do not show the accumulation of amyloid plaques and neurofibrillary tangles that are the core neuropathological features of AD [8, 9]. Studies of brain tissue have even suggested that T2DM is associated with decreases in amyloid plaques and neurofibrillary tangles [10, 11]. So, the link between T2DM and the risk of cognitive impairment may involve other mechanisms.

The role of inflammation in the pathogenesis of T2DM and its associated complications is now well established [12–14]. Recently, a study reported the central role of interleukin (IL-1)-mediated neuroinflammation as a mechanism underlying the cognitive deficits in obesity and diabetes [15]. Microglia are the primary immune effector cells in the brain [16]. In response to brain damage or injury, microglia are activated and undergo morphological as well as functional transformations [17]. Excessive activation of microglia contributes to the progression of chronic neurodegenerative diseases [18], but the underlying mechanisms are still unclear. Recent reports have revealed profound synaptic changes (long-term potentiation and depression) caused by a mirror microglia-mediated inflammatory response in the hippocampus during peripheral organ inflammation [19]. Furthermore, microglia stimulated with lipopolysaccharide phagocytose viable neurons [20]. Studies have shown that the time taken to remove activated microglia determines the severity and duration of central nervous system inflammation.

ATP that supplies energy for cellular metabolism can be released into the extracellular space and play an important role *via* purinergic 2 receptors (P2Rs) in mediating neurotransmission as well as Ca^2+^ waves between astrocytic glial cells [21, 22]. In addition, ATP released locally from damaged tissue induces microgliosis, and activated microglial cells migrate to the site of injury, proliferate, and phagocytose cells and cellular components [23]. Microglial processes in the hippocampal dentate gyrus retract as early as 2 h after injection of lipopolysaccharide and die ~24 h later. These processes are prevented by blocking P2X4 receptors (P2X4Rs), suggesting that P2X4Rs contribute to controlling the fate of activated microglia and their survival [24]. So, early microglial activation mediated by P2X4Rs during neuroinflammation provides a new means of understanding the memory impairment in T2DM.

Taking these findings together, we hypothesized that downregulation of P2X4Rs in the hippocampus leads to the over-activation of microglia and the release of inflammatory mediators, eventually contributing to memory impairment in T2DM rats. Here, we investigated the expression of P2X4Rs in the hippocampus of T2DM rats.

## Materials and Methods

### Animals

Male Sprague-Dawley rats (~250 g) were purchased from the Laboratory Animal Center of Soochow University, China, and housed in a temperature-controlled room at ~23°C with a 12-h day/night cycle (lights on at 08:00 and off at 20:00). The rats were given free access to tap water and food. Relevant studies have shown that a high-fat diet (HFD) followed by a low dose of streptozotocin (STZ) successfully induces T2DM, and the symptoms in this animal model are close to those in patients with T2DM [25], so we used this combination to induce T2DM. Briefly, rats were fed an HFD (20% carbohydrate, 20% protein, and 60% fat) for 2 weeks, followed by one intraperitoneal injection of STZ (30 mg/kg; Sigma, St Louis, MO). Age-matched control rats were fed a normal diet (ND; 60% carbohydrate, 22% protein, and 18% fat), and intraperitoneally injected with citrate buffer. One week after the injection, blood glucose measurements were performed in the morning using blood samples from the tail vein with a glucometer (Johnson & Johnson Medical Co., New Brunswick, NJ). Rats with blood glucose levels ≥16.7 mmol/L were considered diabetic. All experimental procedures were approved by the Animal Care and Use Committee of Soochow University.

### Drug Administration

For insulin tolerance tests, rats were intraperitoneally injected with regular human insulin (Novolin R; Novo Nordisk, Copenhagen, Denmark) at 0.75 U/kg body weight after a 6-h fasting period. The blood glucose level was measured at 0 min, 20 min, 40 min, 60 min, 120 min, and 240 min after the injection.

In the behavioral and molecular experiments, minocycline (Mino, inhibitor of activated microglia, 45 mg/kg body weight) dissolved in normal saline (NS) was intraperitoneally injected daily from 54 days to 61 days after STZ injection [26]. T2DM rats receiving NS injection served as controls.

### Microinjection of Adeno-Associated Virus (AAV)

For behavioral tests and molecular measurements, AAV-NC or AAV-P2X4R from Genechem Co. Ltd (Shanghai, China) was stereotaxically microinjected into the hippocampus of T2DM rats as described in our previous study [22]. Briefly, after the rat was anesthetized by intraperitoneal injection of 4% chloral hydrate, a microinjector was fixed unilaterally to the left side of the skull and aimed at the hippocampus (coordinates with respect to bregma: AP − 4.8 mm, ML 3.2 mm, DV 3.5 mm, angle 0°) or at the right hippocampus. The AAV-NC (8.44 × 10^12^ viral genomes (vg)/mL, 800 nL) or AAV-P2X4R (1.74 × 10^12^ vg/mL, 800 nL) was injected into both the left and right hippocampus 28 days after the STZ injection. After four weeks, rats infected with AAV were assessed in the MWM or sacrificed for further molecular tests.

### Delayed Alternation T-Maze (DAT) Task

Rats were trained on the DAT task using a T-maze device with minor modifications [27]. Behavioral testing was conducted in the daytime between 08:00 and 17:00. Each daily session included one initial trial and nine formal trials. In the initial trial, both arms were baited so that rats would get a food reward by choosing either arm. During the formal trials, rats had to learn to avoid the arm visited in the previous trial and choose the opposite arm to get a reward. If rats made the wrong choice, they would receive a correction procedure to make the correct choice by keeping the baited arm still baited and the procedure would not stop until they visited the baited arm. The errors were counted and divided into two types: a Win-shift failure was defined as wrong choice following a correct choice in the previous trial; and a Lose-shift failure was counted when the rat continued to enter the previously wrong arm. The maze was wiped with alcohol to remove any smell between trials.

### Morris Water Maze (MWM)

This test was carried out with minor modifications as described previously [28]. The training trials were carried out for 5 consecutive days (4 trials daily starting from each quadrant). Each rat was allowed to swim for 60 s. In addition, the rats that failed to locate the platform within the criterion period were placed on it for 5 s. In the probe test trial on day 6, the platform was removed to measure spatial memory. The number of entries into and time spent in the target quadrant where the platform was previously located, and the mean distance from the previous platform position for each rat were recorded by Anymaze video tracking system software (Global Biotech Inc., Shanghai, China).

### Immunofluorescence Assay

Rats were deeply anesthetized by intraperitoneal injection of 4% chloral hydrate, then perfused transcardially with 300 mL NS followed by precooled 4% paraformaldehyde (PFA). The brain was rapidly removed, post-fixed in PFA for 3 h, and then immersed in phosphate-buffered sucrose to dehydrate gradiently. For double labeling, 14-μm sections were simultaneously incubated with antibodies against P2X4Rs (1:100, Alomone Labs, Jerusalem, Israel; APR-002) and NeuN (1:50, Merk Millpore, Darmstadt, Germany; MAB377) or GFAP (1:300, Cell Signaling Technology, Danvers, MA; 3670S) or CD11b (1:100, Bio-Rad, Berkeley, CA; MCA275R) at 4°C overnight and then with Alexa Fluor 488 (1:500, Molecular Probes, Shanghai, China; A21206) and 555 (1:100, Molecular Probes, A31570) for 2 h at room temperature (RT). The primary antibodies were omitted for negative controls. After staining, images were captured on an Axioscope A1 microscope (Zeiss, Jena, Thuringian, Germany). The fluorescent cells were counted and analyzed using ImageJ software (Rawak Software Inc., Stuttgart, Baden-Württemberg, Germany).

### Western Blotting

Each rat was guillotined and the brain was quickly dissected out and lysed by ultrasonication in an ice bath. Twenty micrograms of total protein was fractionated on 10% polyacrylamide gel. Proteins were then transferred to PVDF membranes for 2 h at 200 mA in an ice bath. The membranes were blocked in 5% non-fat milk powder at RT for 2 h and then incubated with primary antibodies (GAPDH, 1:1000, Hangzhou Goodhere Biotechnology, AB-P-R001; P2X4Rs, 1:500, Alomone Labs, APR-002; and ATM, 1:100, Santa Cruz, CA; sc-23921) at 4°C overnight. Then, the membranes were incubated with HRP-conjugated secondary antibodies (GAR0072 or GAM0072, 1:2000, Multi Science, Hangzhou, China) at RT. Bands were visualized using an enhanced chemiluminescence detection kit for HRP (EZ-ECL, Biological Industries, Kibbutz Beit Haemek, Israel; 20-500-120) and appropriately exposed in a chemiluminescence imaging system (ChemiDoc XRS, Bio-Rad, Hercules, CA). Band intensities were measured using ImageJ. Protein expression was normalized to GAPDH.

### Real-Time Quantitative Polymerase Chain Reaction (qPCR)

Total RNA was exacted from the hippocampus using the TRIzol method. cDNA was synthesized from total RNA using the EasyScript First-Strand cDNA Synthesis SuperMix kit (Transgen Biotech, Beijing, China) following the manufacturer’s instructions. The primer of *p2x4r* used in qPCR was: Forward 5′-GGCTACCAGGAAACGGACTC-3′ and Reverse 5′-ATCACATAGTCCGCCACGTC-3′. Negative control reactions were performed by omitting the cDNA template. The relative expression level for each target gene was normalized using the 2^−ΔΔCt^ method.

### Long Amplicon PCR (LA-PCR)

Genomic DNA was extracted from the hippocampus of T2DM and control rats using the QIAamp DNA Mini kit according to the manufacturer’s instructions (Qiagen, Dusseldorf, Germany). Briefly, ground hippocampal tissue was digested in proteinase K at 56°C for 3 h, then total DNA was purified using the QIAamp procedures. NCBI GenBank showed that the *p2x4r* gene contains 17,652 base pairs. Long- and short-PCR were separately performed using Phanta Max Master Mix (Vazyme Biotech Co., Ltd, Nanjing, China). The primers were as follows: *Long-p2x4r* F: 5′-GAGCCTGCCGGAGCTGGTGGGTGGA-3′, R: 5′-TGCTTGGGCAACCCTGAGTATTTGTGGAGT-3′; *Short-p2x4r* F: 5′-GACGAGCAAATAACTAAGCC-3′, R: 5′-TGTTTCCCTGTAATCCACT-3′. The long PCR procedure was conducted as follows: 95°C, 10 min; 95°C, 15 s, 76°C, 18 min for 3 cycles; 95°C, 15 s, 75°C, 18 min for 3 cycles; 95°C, 15 s, 74°C, 18 min for 3 cycles; 95°C, 15 s, 73.6°C, 18 min for 24 cycles; 72°C, 10 min. Agarose gel electrophoresis was carried out after long- and short-PCR. The densitometry of bands was determined using ImageJ, and the long-PCR/short-PCR ratio was used for statistical analyses.

### Statistical Analysis

All values are shown as the mean ± SEM. Data were analyzed using OriginPro 8 (OriginLab, Northampton, MA) and Prism 6 (Graph Pad, San Diego, CA) softwares. Gaussian distribution tests were conducted before analysis. The two-sample *t*-test or Mann-Whitney test was used to determine the significance of differences between two groups. Two-way ANOVA followed by Tukey’s *post hoc* test was performed when appropriate. *P* < 0.05 was considered statistically significant.

## Results

### T2DM Rats Exhibit Memory Impairment

**Fig. 1:**
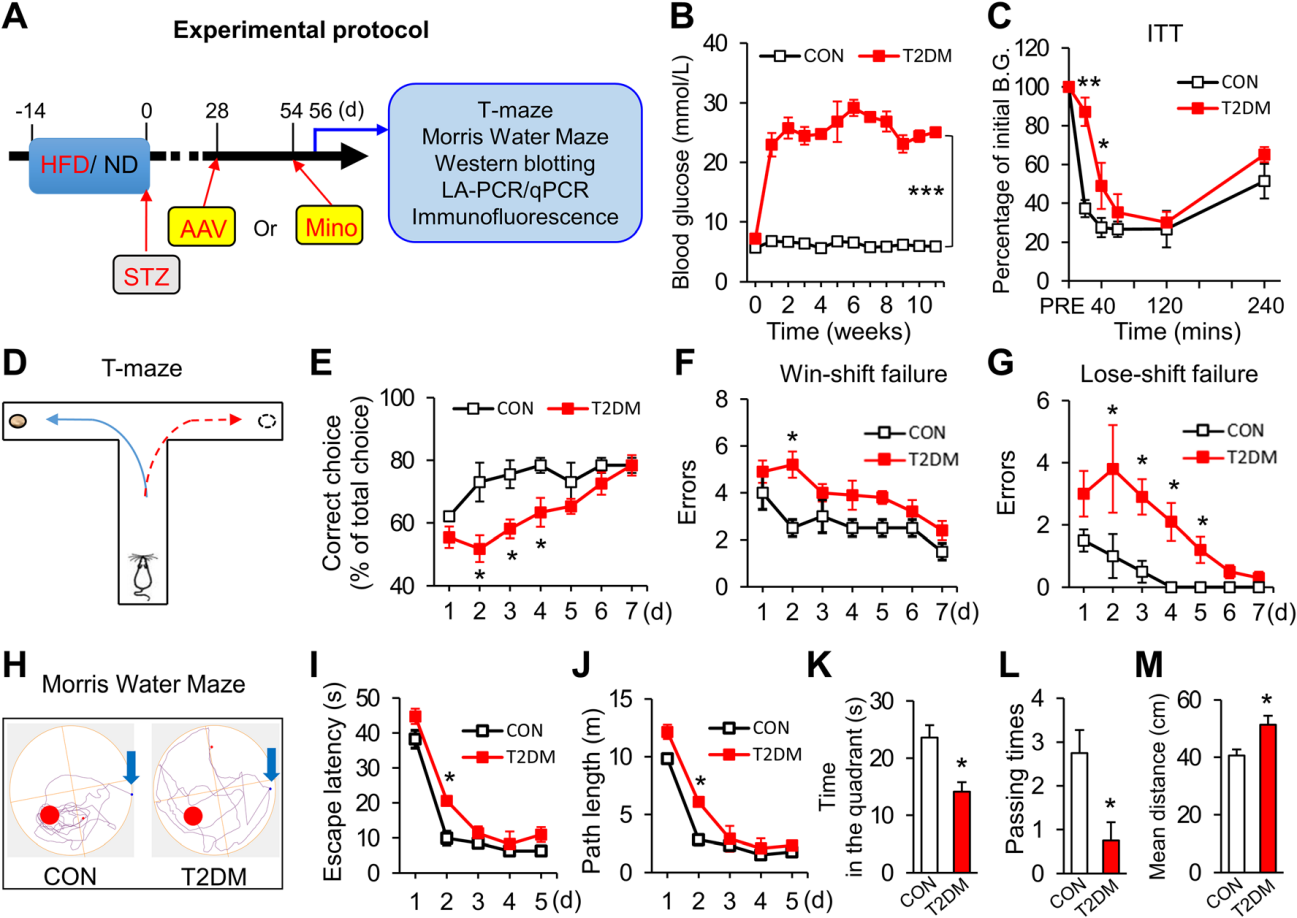
T2DM rats show memory impairment. **A** Experimental protocol. Rats were fed an HFD or ND for two weeks then intraperitoneally injected with STZ or NS. AAV-NC or AAV-P2X4R was microinjected into the hippocampus on day 28. Minocycline or NS was injected daily for one week from day 54. On day 56, behavioral tests and molecular determinations were conducted (HFD, high-fat diet; ND, normal diet; STZ, streptozotocin; AAV, adeno-associated virus; Mino, minocycline). **B** Blood glucose levels in T2DM rats and controls (CON) (*n* = 8/group; ***P < 0.001 *vs* CON, two-way ANOVA). **C** Insulin tolerance test (ITT) results showing the percentage of initial blood glucose (B.G.) in T2DM and CON rats after intraperitoneal insulin injection (*n* = 8/group; *P < 0.05, **P < 0.01 *vs* CON, two-way ANOVA followed by Tukey’s *post hoc* test). **D** The T-maze consisted of a stem (50 cm × 10 cm) and two arms (40 cm × 10 cm) with walls 20 cm high. **E** Correct choices of T2DM and CON rats in the T-maze (*n* = 8/group; *P < 0.05 *vs* CON, two-way ANOVA followed by Tukey’s *post hoc* test). **F** Errors using the Win-shift strategy by T2DM and CON rats (*n* = 8/group; *P < 0.05 *vs* CON, two-way ANOVA followed by Tukey’s *post hoc* test). **G** Errors using the Lose-shift strategy by T2DM and CON rats (*n* = 8/group; *P < 0.05 *vs* CON, two-way ANOVA followed by Tukey’s post *hoc test*). **H** Representative tracking of T2DM and CON rats in the MWM test on day 6. **I**, **J** Escape latency and path length of T2DM and CON rats in training trials of the MWM (*n* = 16/group; *P < 0.05 *vs* CON, two-way ANOVA followed by Tukey’s *post hoc* test). **K**–**M** Time in the target quadrant, passing times, and mean distance from the platform of T2DM and CON rats in the probe test on day 6 (*n* = 16/group; *P < 0.05, two-sample *t*-test for **K** and **M**, Mann-Whitney test for **L**).

To induce T2DM, rats were fed the HFD for 2 weeks followed by an intraperitoneal injection of a low dose of STZ. Rats fed the normal diet and intraperitoneally injected with NS were used as controls (Fig. 1A). One week after STZ injection, the blood glucose levels of T2DM rats were >16.7 mmol/L and lasted to the 11th week while they were normal in control rats (Fig. 1B). Confirming the induction of T2DM, insulin tolerance tests showed that the blood glucose levels of T2DM rats were significantly higher than those of controls 20 min and 40 min after intraperitoneal injection of insulin (0.75 U/kg), which means that these rats were insulin tolerant (Fig. 1C).

Then we investigated how T2DM rats performed in the DAT task (Fig. 1D). In a week of trials, the correct choice rate of T2DM rats was significantly lower than that of control rats on days 2–4, but there was no significant difference on days 5–7 (Fig. 1E). Interestingly, analysis of error types revealed that T2DM rats showed more errors using the Win-shift strategy on day 2, and they made many more errors using the Lose-shift strategy on days 2–5 than controls (Fig. 1F, G). T2DM rats failed to use the Win-shift and Lose-shift strategies in the DAT task, which led to their low correct choice rate. These results indicated that T2DM rats have a deficit in spatial working memory.

To further confirm the memory impairment of T2DM rats, we applied the MWM test and there was no significant difference between T2DM rats and controls in the learning procedure from day 1 to day 5, except for day 2 (Fig. 1I, J). However, in the probe test on day 6, the time that T2DM rats remained in the quadrant where the platform had been located was greatly reduced compared with controls (Fig. 1K). Similarly, the passing times of T2DM rats were markedly fewer than in controls (Fig. 1L). Furthermore, the mean distance from the platform was remarkably greater in T2DM rats than in controls (Fig. 1M). These data confirmed that T2DM rats exhibit memory impairment.

### Microglia are Activated in the Hippocampus of T2DM Rats

**Fig. 2:**
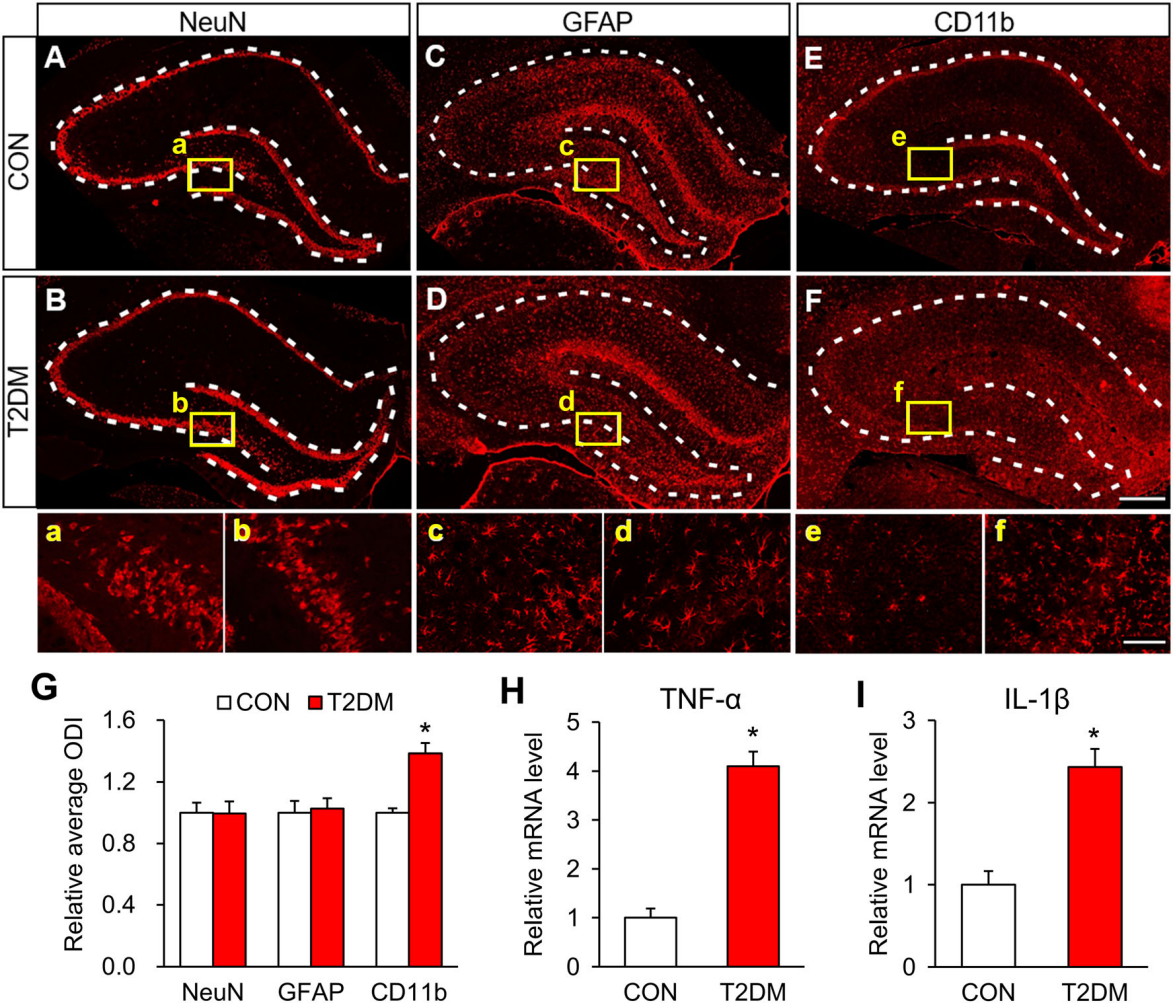
Microglia are activated in the hippocampus of T2DM rats. **A**–**F** In the hippocampus of T2DM and control (CON) rats: NeuN-labeled neurons (**A, B, a, b**); GFAP-labeled astrocytes (**C, D, c, d**); and CD11b-labeled microglia (**E, F, e, f**) (scale bars, 500 μm in **F**; 100 μm in **f**). Dashed lines outline the hippocampus. **G** Relative numbers of NeuN-, GFAP-, and CD11b-positive cells in the hippocampus of T2DM and CON rats (*n* = 4/group; *P < 0.05 *vs* CON, two-sample *t*-test). **H**, **I** The mRNA levels of TNF-α (**H**) and IL-1β (**I**) in the hippocampus of T2DM and CON rats (*n* = 4/group; *P < 0.05 *vs* CON, two-sample *t*-test).

We next investigated whether the functions of cell types in the hippocampus were altered in T2DM rats. Immunofluorescence assays showed that the number of neurons labeled by NeuN in the hippocampus of T2DM rats did not differ from controls (Fig. 2A, B). Similarly, the number and appearance of astrocytes marked by GFAP did not differ in the hippocampus of T2DM rats (Fig. 2C, D). However, and interestingly, the number of CD11b-stained microglia was dramatically higher in T2DM rats than in controls (Fig. 2E–G). And these microglia in T2DM rats were amoeboid in shape (Fig. 2F). Since activated microglia synthesize and secrete inflammatory mediators [29], we assessed the expression of TNF-α and IL-1β in the hippocampus using qPCR. The results showed that their mRNA levels were significantly higher in T2DM rats than in controls (Fig. 2H, I). These data demonstrated that microglia are clearly activated in the hippocampus of T2DM rats.

### Minocycline Treatment Inhibits Activated Microglia and Improves the MWM Performance of T2DM Rats

**Fig. 3:**
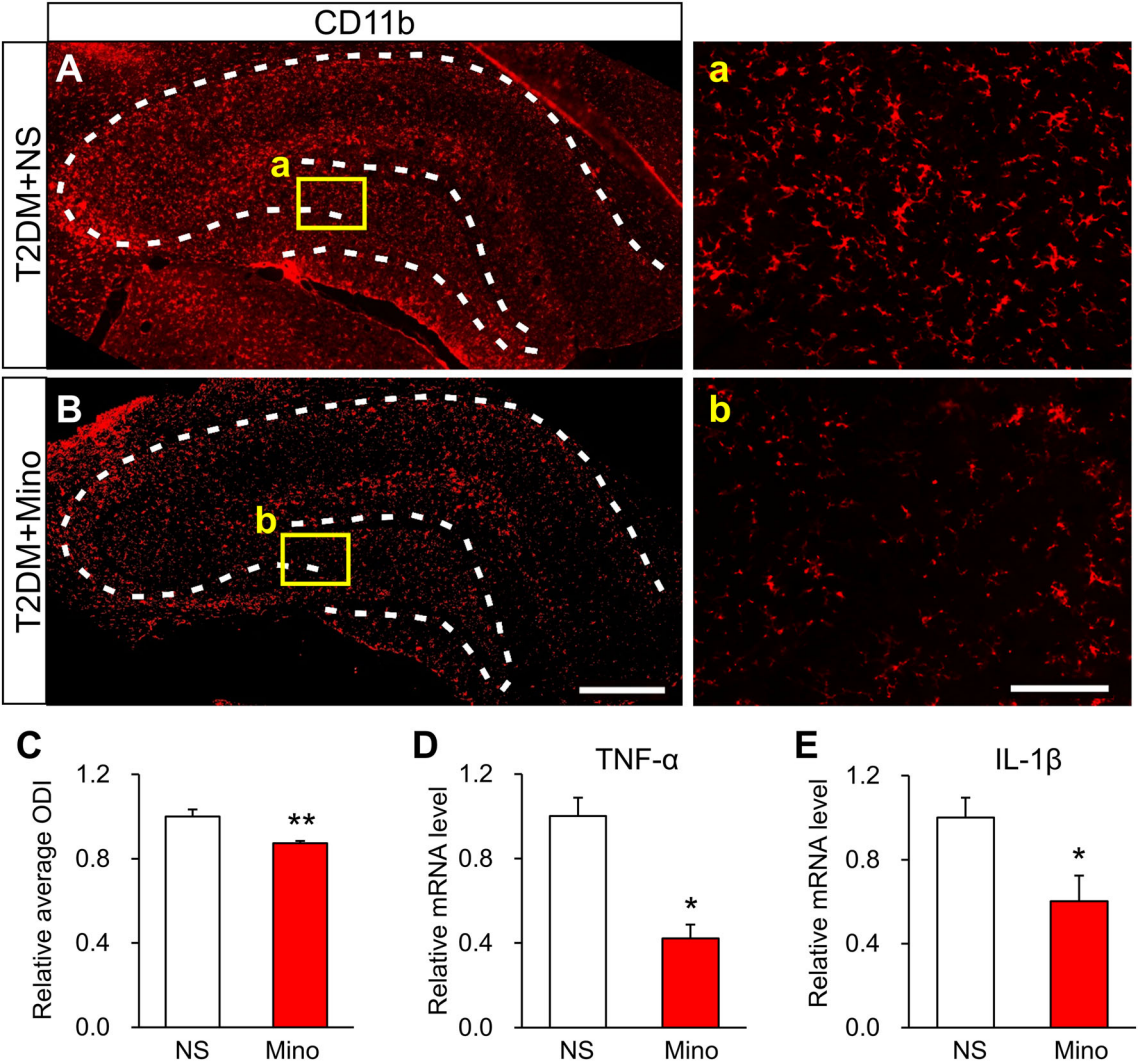
Minocycline treatment inhibits activated microglia. **A**–**C** Representative images and statistics for the number of activated microglia labeled by CD11b in the hippocampus of T2DM rats injected with minocycline (Mino) or normal saline (NS) (scale bars, 500 μm in **B**; 100 μm in **b**; *n* = 4/group; **P < 0.01 *vs* NS, two-sample *t*-test). Dashed lines outline the hippocampus. **D**, **E** TNF-α and IL-1β levels after Mino or NS injection (*n* = 4/group; *P < 0.05 *vs* NS, two-sample *t*-test).

Minocycline is an effective inhibitor of activated microglia [30]. Using immunofluorescence assays, we showed that activated microglia in the hippocampus of T2DM rats were markedly reduced by daily intraperitoneal injections of minocycline for a week from day 54 (Fig. 3A–C). In addition, the mRNA levels of TNF-α and IL-1β in the hippocampus of T2DM rats were significantly decreased compared with NS injection (Fig. 3D, E). These data indicated that minocycline effectively inhibits the activated microglia in T2DM rats.

**Fig. 4:**
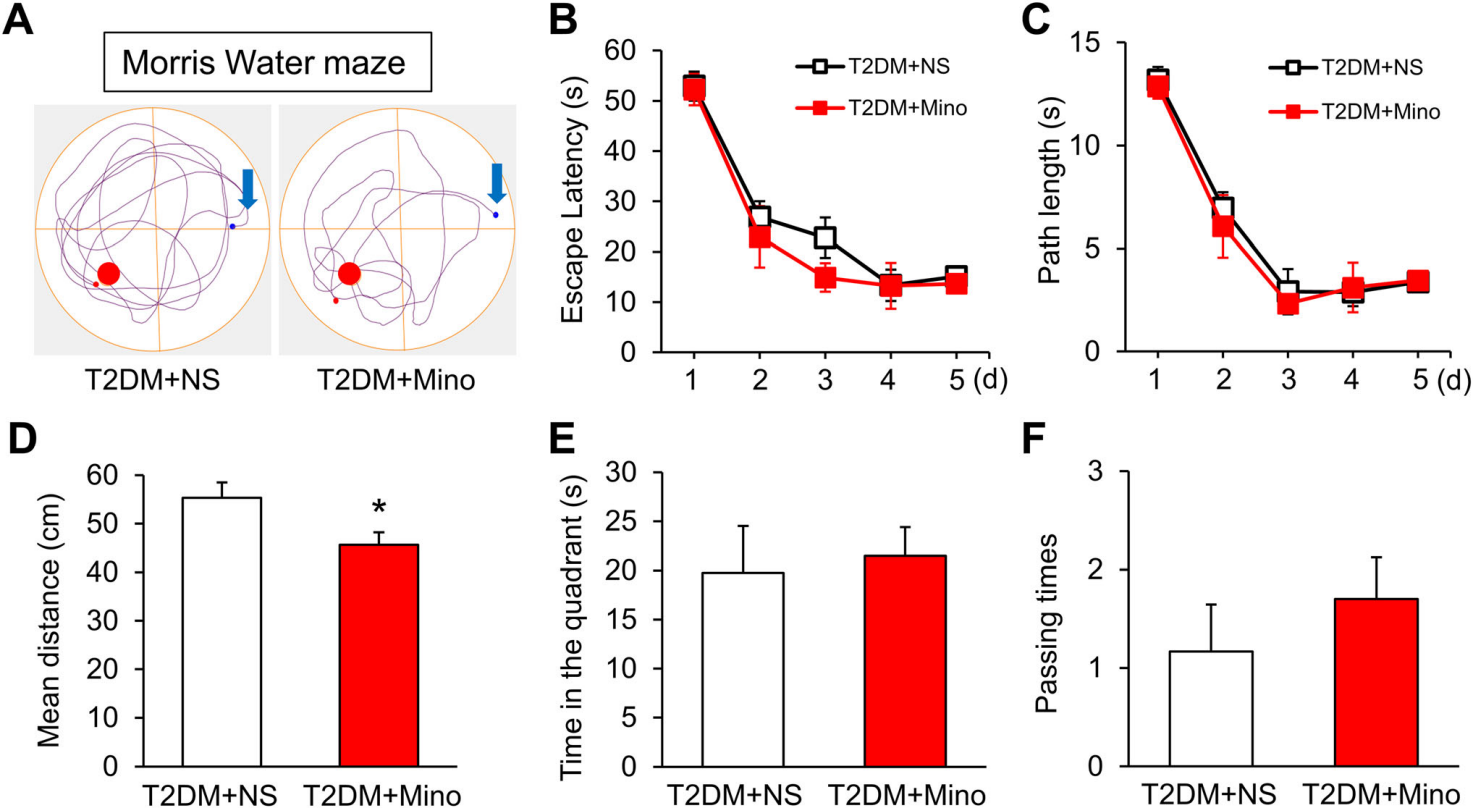
Minocycline improves the MWM performance of T2DM rats. **A** Tracking of T2DM rats after injection of normal saline (NS) or minocycline (Mino) on day 6. **B**, **C** Escape latency and path length of T2DM rats injected with minocycline or NS (*n* = 4/group; no significant difference, two-way ANOVA followed by Tukey’s *post hoc* test). **D** Average distance from the platform of T2DM rats injected with minocycline or NS (*n* = 8/group; *P < 0.05 *vs* T2DM+NS, two-sample *t*-test). **E**, **F** Time in the platform quadrant and passing times of T2DM rats injected with minocycline or NS (*n* = 8/group; no significant difference, two-sample *t*-test for** E** and Mann–Whitney test for** F**).

We then determined the effect of minocycline on the performance of T2DM rats in the MWM. There was no difference between T2DM rats treated with minocycline and NS during the learning period (Fig. 4B, C). In the probe test on day 6, we found that minocycline significantly reduced the mean distance traveled by T2DM rats compared with the NS group (Fig. 4D), although the time spent in the target quadrant and crossing times did not differ (Fig. 4E, F). Besides, T2DM rats with minocycline appeared to better memorize the target position than the NS group (Fig. 4A). These results indicated that minocycline rescues the spatial memory impairment in T2DM rats.

### P2X4Rs are Down-Regulated in the Hippocampus of T2DM Rats

**Fig. 5:**
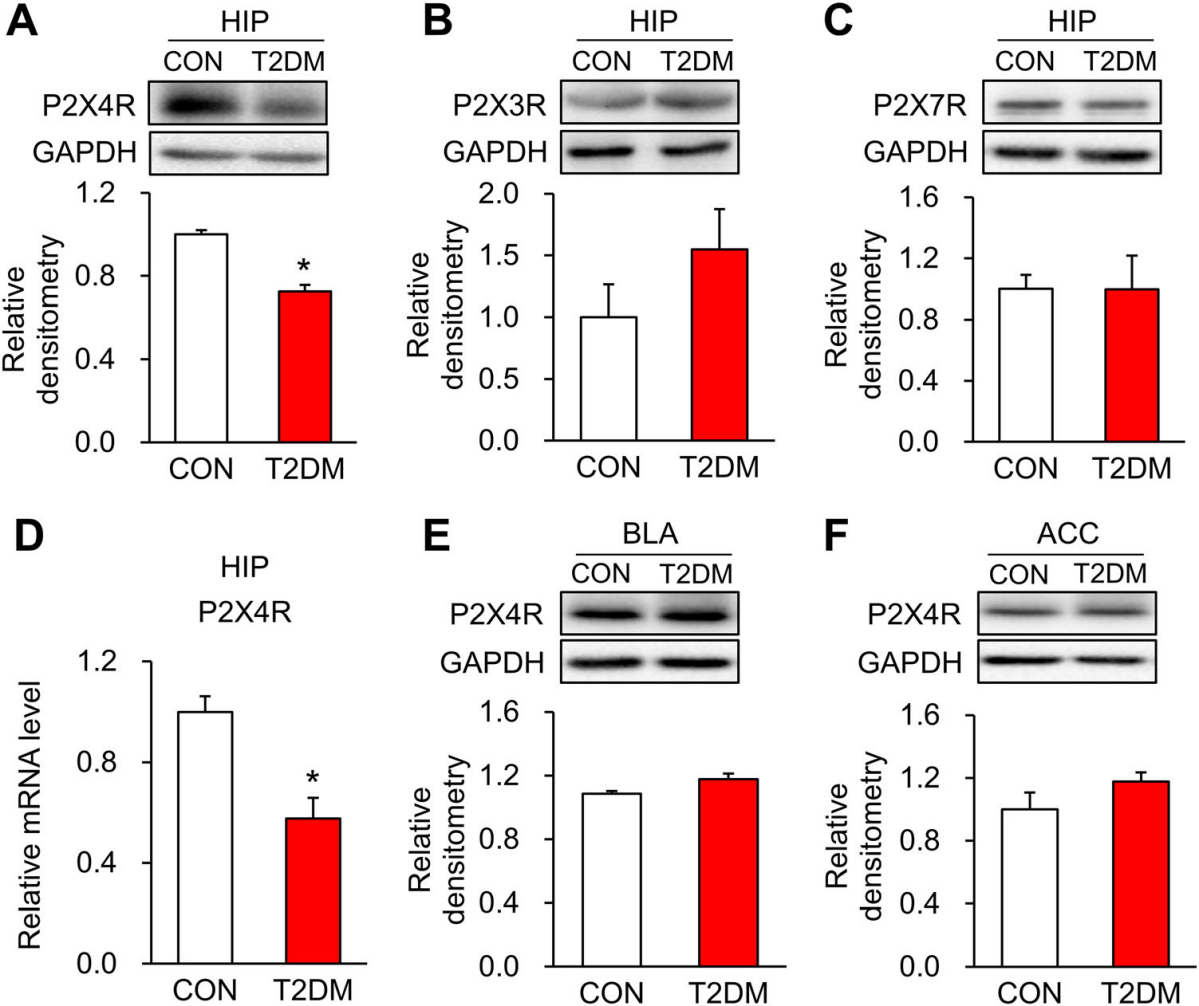
P2X4R expression is down-regulated in the hippocampus of T2DM rats. **A** Protein expression of P2X4Rs in the hippocampus of T2DM and control (CON) rats (*n* = 4; *P < 0.05 *vs* CON, two-sample *t*-test). **B**, **C** Protein expression of P2X3Rs and P2X7Rs in the hippocampus of T2DM and CON rats (*n* = 4/group; no significant difference, two sample *t*-test). **D** mRNA levels of P2X4Rs in the hippocampus of T2DM and CON rats (*n* = 4/group; *P < 0.05 *vs* CON, two-sample *t*-test). **E**, **F** Protein expression of P2X4Rs in the basolateral amygdaloid nucleus (BLA) and anterior cingulate cortex (ACC) of T2DM and CON rats (*n* = 4/group, no significant difference, two-sample *t*-test).

Here we showed that P2X4R expression was significantly decreased at both the protein and mRNA levels in the hippocampus of T2DM rats when compared with control rats (Fig. 5A, D), while the protein levels of P2X3Rs and P2X7Rs were not altered (Fig. 5B, C). In addition, P2X4R expression in the basolateral amygdaloid nucleus (BLA) and anterior cingulate cortex (ACC) did not significantly differ between T2DM rats and controls (Fig. 5E, F).

### P2X4Rs are Mainly Expressed in Microglia of the Hippocampus

**Fig. 6:**
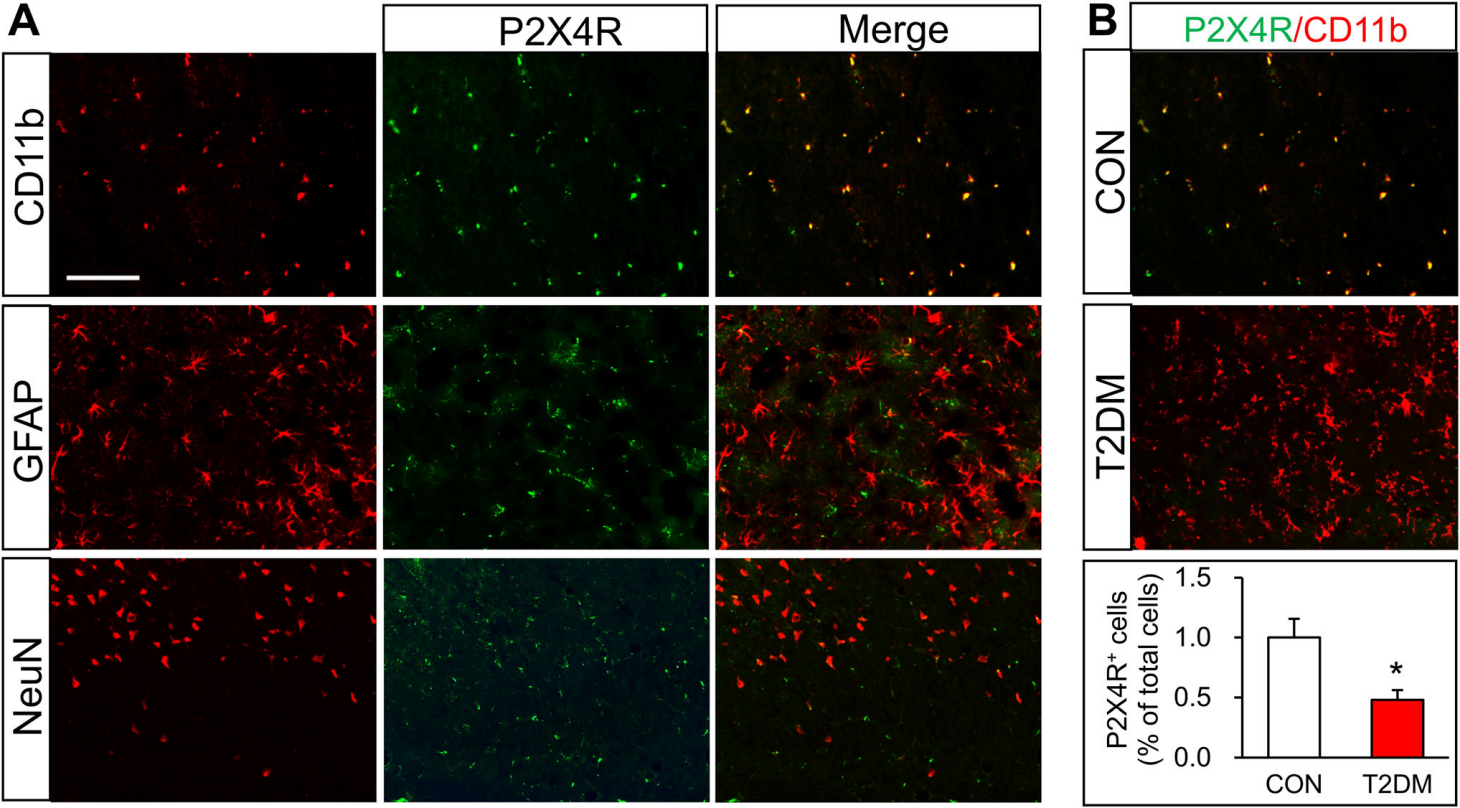
P2X4Rs are mainly expressed in microglia in the hippocampus. **A** Representative images of P2X4Rs (green) co-expressed with CD11b (red), but not with GFAP (red) or NeuN (red) (scale bar, 100 μm). **B** Representative images and statistics showing the percentages of P2X4R-positive cells in T2DM and control (CON) rats (*n* = 4/group; *P < 0.05 *vs* CON, two-sample *t*-test).

Since previous studies have shown that P2X4Rs are predominantly expressed in microglia in the spinal cord [31], we performed an immunofluorescence study to determine the location of P2X4Rs in the hippocampus of control rats. We found that P2X4Rs were mainly co-expressed with CD11b, the marker of microglia, but not with GFAP or NeuN (Fig. 6A). In addition, we analyzed P2X4R expression in the hippocampal microglia of both T2DM and control rats. The immunofluorescence results showed that the co-expression of P2X4Rs and CD11b was dramatically lower in T2DM rats than in controls (Fig. 6B). These results implied that P2X4R expression is decreased in the microglia of the hippocampus in T2DM rats.

### DNA Damage Contributes to the Down-Regulation of P2X4Rs

**Fig. 7:**
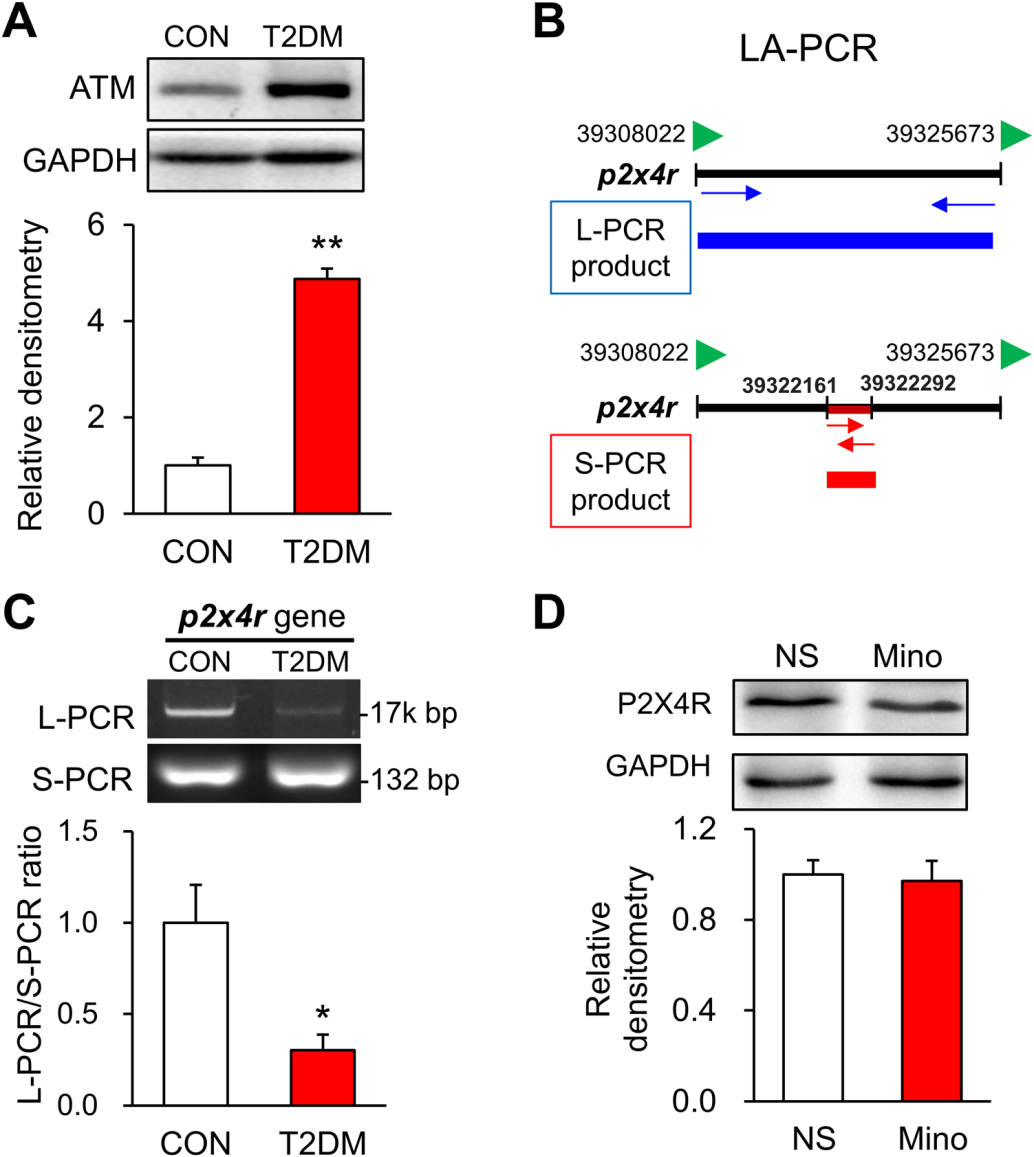
T2DM results in DNA damage of the *p2x4r* gene. **A** Expression of ATM in the hippocampus of T2DM and control (CON) rats (*n* = 3/group; **P < 0.01 *vs* CON, two-sample *t*-test). **B** Diagram showing that Long Amplicon PCR (LA-PCR) contains the Long-PCR product and the Short-PCR product. **C** L-PCR/S-PCR ratio of *p2x4r* gene in the hippocampus of T2DM and CON rats (*n* = 4/group; *P < 0.05 *vs* CON, two-sample *t*-test). **D** Protein expression of P2X4R after minocycline (Mino) and normal saline (NS) treatment (*n* = 4 for NS group, *n* = 3 for Mino group; *P* > 0.05, two-sample *t*-test).

We next investigated the mechanisms by which P2X4R expression is down-regulated. Since DNA damage is involved in patients with T2DM [32], we determined whether DNA damage contributes to the downregulation of P2X4R expression. Western blotting showed that ATM (ataxia telangiectasia mutated), a marker of DNA damage, was significantly up-regulated in the hippocampus of T2DM rats (Fig. 7A). LA-PCR technology was used to further detect DNA damage in specific genes (Fig. 7B). The Long-chain PCR/Short-chain PCR ratio of the *p2x4r* gene was significantly reduced in the hippocampus of T2DM rats when compared with control rats (Fig. 7C). These data indicated that DNA damage of the *p2x4r* gene contributes to the decreased P2X4R expression. In addition, minocycline injection did not affect P2X4R expression in the hippocampus of T2DM rats compared with NS injection (Fig. 7D), suggesting that decreased P2X4Rs might be an upstream signal of activated microglia.

### Overexpression of P2X4Rs Reduces the Activation of Microglia

**Fig. 8:**
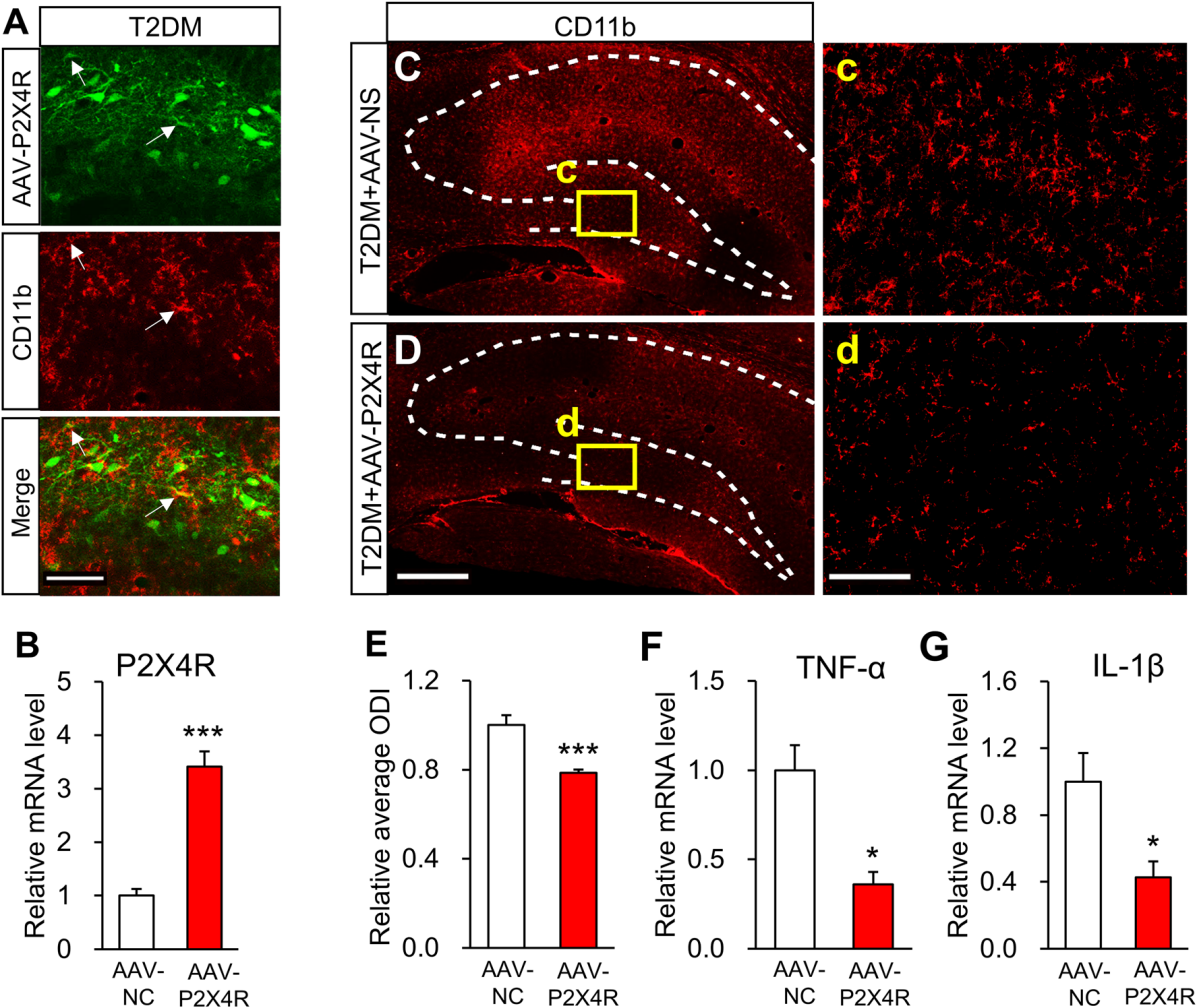
Overexpression of P2X4Rs blocks the activation of microglia. **A** Representative images of AAV-P2X4R (green) and CD11b (red) staining in the hippocampus of a T2DM rat (arrows, co-localization of AAV-P2X4R and CD11b; broken lines outline the hippocampus). **B** P2X4R expression after microinjection of AAV-P2X4R or AAV-NC (*n* = 4/group; ***P < 0.001 *vs* AAV-NC, two-sample *t*-test). **C**–**E** Representative images and statistics of activated microglia labeled by CD11b in the hippocampus of T2DM rats injected with AAV-P2X4R or AAV-NC (scale bars, 500 μm in **D**, 100 μm in **d**; *n* = 4/group; ***P < 0.001 *vs* AAV-NC, two-sample *t*-test). **F**, **G** TNF-α and IL-1β mRNA levels after AAV-P2X4R injection (*n* = 4/group; *P < 0.05 *vs* AAV-NC, two-sample *t*-test).

To further investigate the involvement of P2X4Rs in microglial activation in T2DM rats, we overexpressed P2X4Rs in the bilateral hippocampus of these rats by microinjection of AAV-P2X4R and demonstrated that P2X4Rs were successfully overexpressed in the hippocampus (Fig. 8A, B). Importantly, after overexpression of P2X4Rs, activated microglia were markedly suppressed in the hippocampus of T2DM rats (Fig. 8C–E). And the mRNA levels of TNF-α and IL-1β were also significantly reduced when P2X4Rs were overexpressed (Fig. 8F, G). Above all, these data indicated that P2X4R overexpression can effectively inhibit activated microglia.

### Overexpression of P2X4Rs Rescues Memory in T2DM Rats

**Fig. 9:**
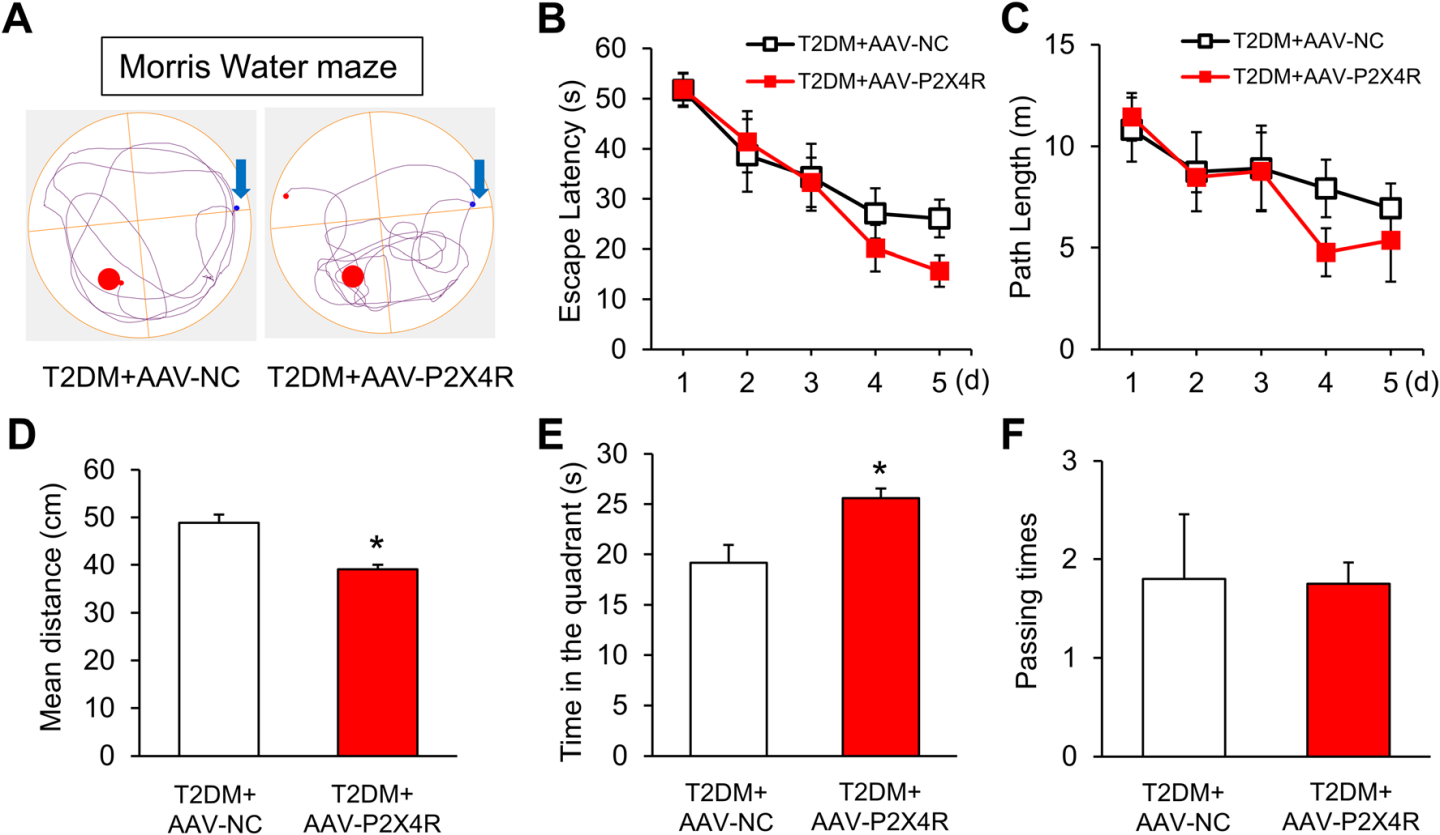
P2X4R overexpression rescues memory in T2DM rats. **A** Tracking of T2DM rats injected with AAV-NC or AAV-P2X4R on day 6 in the MWM test. **B**, **C** Escape latency and path length in the MWM training trials of the two groups (*n* = 8/group; no significant difference, two-way ANOVA followed by Tukey’s *post hoc* test). **D** Mean distance from the platform of T2DM rats injected with AAV-P2X4R or AAV-NC in the probe test on day 6 (*n* = 8/group; *P < 0.05 *vs* AAV-NC, two-sample *t*-test). **E** Time in the target quadrant of T2DM rats injected with AAV-P2X4R or AAV-NC (*n* = 8/group; *P < 0.05 *vs* AAV-NC, two-sample *t*-test). **F** Passing times of the two groups (*n* = 8/group; no significant difference, Mann-Whitney test).

Four weeks after microinjection of AAV-P2X4R, we assessed rat behaviors in the MWM. Tracking showed that T2DM rats injected with AAV-P2X4R moved around the platform position, but those injected with AAV-NC did not improve (Fig. 9A). Although the two groups did not differ in the learning exercise (Fig. 9B, C), the mean distance from the platform was remarkably lower in T2DM rats injected with AAV-P2X4R than in those injected with AAV-NC (Fig. 9D). In contrast, the time T2DM rats injected with AAV-P2X4R remained in the quadrant with the platform was longer than that of those injected with AAV-NC (Fig. 9E), while the passing times did not improve (Fig. 9F). These data demonstrated that P2X4R overexpression markedly improves the spatial memory of T2DM rats.

## Discussion

T2DM is associated with an increased risk of AD and vascular dementia induced by increasing oxidative stress, inflammation, impaired insulin, and amyloid metabolism [33]. Here, we showed that T2DM rats exhibited clear spatial memory impairment in the MWM and the DAT task, because they had fewer passing times and spent less time in the platform quadrant but traveled a greater distance than controls in the MWM probe test on day 6. However, there was no significant difference between T2DM rats and controls for finding the platform in the training period. One possible reason is that the diabetic rats might be in the early stages of cognitive dysfunction; this differs from patients who have suffered from diabetes for 10 years on average. In addition, greater learning intensity and density led to faster learning by control and T2DM rats, because the time they needed to find the platform both declined to ~10 s on days 3–5 (Fig. 1I). It has been established that memory is strengthened in the hippocampus by the consolidation of new synaptic connections with the repeated learning of information [34]. So, it is possible that in the learning and training procedure, the hippocampus of T2DM rats produces new synaptic connections and temporary short-term memory, but these connections are not stable and long-term memory cannot be stored correctly.

In line with memory impairment, the P2X4R expression was significantly decreased at both the transcriptional and translational levels in the hippocampus of T2DM rats. The downregulation of P2X4R expression in the hippocampus seemed to be specific since there was no alteration in the expression of P2X4Rs in the BLA and ACC. These data suggested that P2X4Rs might be important in the development of the memory impairment of T2DM rats. This is consistent with other reports showing stronger long-term potentiation in the hippocampus of wild-type mice than that in P2X4R-knockout mice [35, 36]. ATM, an important marker of DNA damage, was pronouncedly increased in the hippocampus of T2DM rats. So, we determined whether DNA damage is responsible for the P2X4R downregulation. Several methods can be used to detect DNA damage, such as the γH2AX signal [37], but they are all global tests. We used LA-PCR, a newly-developed technology, to precisely detect DNA damage on one specific gene [38]. Because a long fragment of nucleotides is more easily damaged than a short one, we designed specific primers for long and short fragments of the *p2x4r* gene. As expected, the long-PCR amplification of the *p2x4r* gene in the hippocampus of T2DM rats was significantly less than that in controls. These data strongly suggested that DNA damage of the *p2x4r* gene occurs in T2DM rats. However, how this damage happened was not clear. Accumulating studies have reported that oxidative stress can induce DNA damage [39–41]. We also found that the concentration of reactive oxygen species was dramatically increased in hippocampal neurons after 24 h of culture with high glucose (Fig. S1), indicating that diabetes may cause oxidative stress that leads to DNA damage. Above all, we demonstrated here that DNA damage is a novel regulatory mechanism of P2X4R expression in the hippocampus of T2DM rats.

Since the mRNA level of P2X4Rs was significantly reduced in the hippocampus of T2DM rats (Fig. 5D), transcription of the *p2x4r* gene was inhibited. DNA methylation is one of the most common epigenetic mechanisms in the regulation of gene expression [42]. Our previous study showed that the expression of purinergic P2X3Rs is regulated by DNA methylation [43, 44]. Therefore, we took the methylation status of the *p2x4r* gene DNA into account. However, there were no CpG islands in the promoter of the *p2x4r* gene (Fig. S2). We therefore can exclude the involvement of DNA methylation and demethylation in the down-regulation of P2X4R expression in the hippocampus of T2DM rats. It is worth noting that other mechanisms of epigenetic regulation such as histone modification or non-coding RNAs need to be investigated in future.

Using immunofluorescence assays, we showed that P2X4Rs were specifically expressed in hippocampal microglia, indicating a role of microglia in the memory impairment of T2DM rats. Although the expression of P2X4Rs was decreased, the microglia were strongly activated in the hippocampus of T2DM rats. Once activated, microglia secrete a variety of inflammatory factors and we found that TNF-α and IL-1β were significantly increased in the hippocampus of T2DM rats (Fig. 2H, I). Treatment with minocycline, a microglia inhibitor, significantly reduced microglial activation and the expression of TNF-α and IL-1β (Fig. 3D, E), further supporting a role of microglia. More importantly, the mean distance from the platform after minocycline treatment was markedly decreased in T2DM rats (Fig. 4D). Therefore, over-activated microglia in the hippocampus are indeed involved in the memory impairment of T2DM rats. Microglia have the capacity to release a large number of substances that can be detrimental to surrounding neurons, including glutamate, ATP, and reactive oxygen species. However, how altered neurotransmission following acute insults or chronic neurodegenerative conditions modulates neuronal functions is still poorly understood [23]. Besides, microglia have been demonstrated to play opposing roles in the regulation of synaptic plasticity in the spinal cord and hippocampus [45–47]. Indeed, microglia show a variety of different properties even in the sub-nuclei of the basal ganglia [48]. This warrants further investigation into the detailed mechanism by which over-reaction of microglia induces memory impairment in T2DM rats.

In order to investigate whether the microglial over-reaction is mediated by downregulation of P2X4R expression, we designed two crucial experiments. First, we overexpressed P2X4Rs by AAV microinjection into the hippocampus of T2DM rats, and found that AAV co-localized with some CD11b-positive microglia in addition to neurons (Fig. 8A). Although we cannot exclude an exogenous role of P2X4R in neurons, we showed for the first time that overexpression of P2X4Rs dramatically suppressed the microglial activation and the mRNA levels of TNF-α and IL-1β in the hippocampus of T2DM rats, indicating that the P2X4R is an upstream signaling molecule in the regulation of microglial activation. Encouraged, we performed the second experiment to further confirm this by examination of P2X4R expression after administration of minocycline. Since minocycline did not affect the expression of P2X4Rs, it is reasonable to assume that downregulation of P2X4R expression might be an upstream mechanism underlying the activation of microglial cells. This is consistent with the previous report that P2X4Rs determine the fate of microglia, especially in the early stage of controlling the apoptosis of activated microglia [24]. Our findings also suggest that decreased P2X4Rs are not able to regulate the apoptosis of activated microglia, which results in their excessive activation. However, the mechanism of suppression of activated microglia by P2X4R overexpression is elusive. Conversely, overexpression of P2X4Rs remarkably improved the memory of T2DM rats. This is a very important finding that provides strong evidence for the development of novel strategies for the treatment of memory disorders. Recently, reports have shown that P2X4-deficient mice exhibit sociocommunicative and sensorimotor impairments [49], and that P2X4Rs favor remyelination in autoimmune encephalitis [50]. Taken together, our findings highlight a putative role of P2X4Rs in the regulation of memory functions in T2DM patients. In addition, P2Y12R is a microglial signature gene and participates in normal brain function and diseases [51–53]. Whether P2Y12R contributes to the memory impairment of T2DM rats deserves further investigation.

**Fig. 10:**
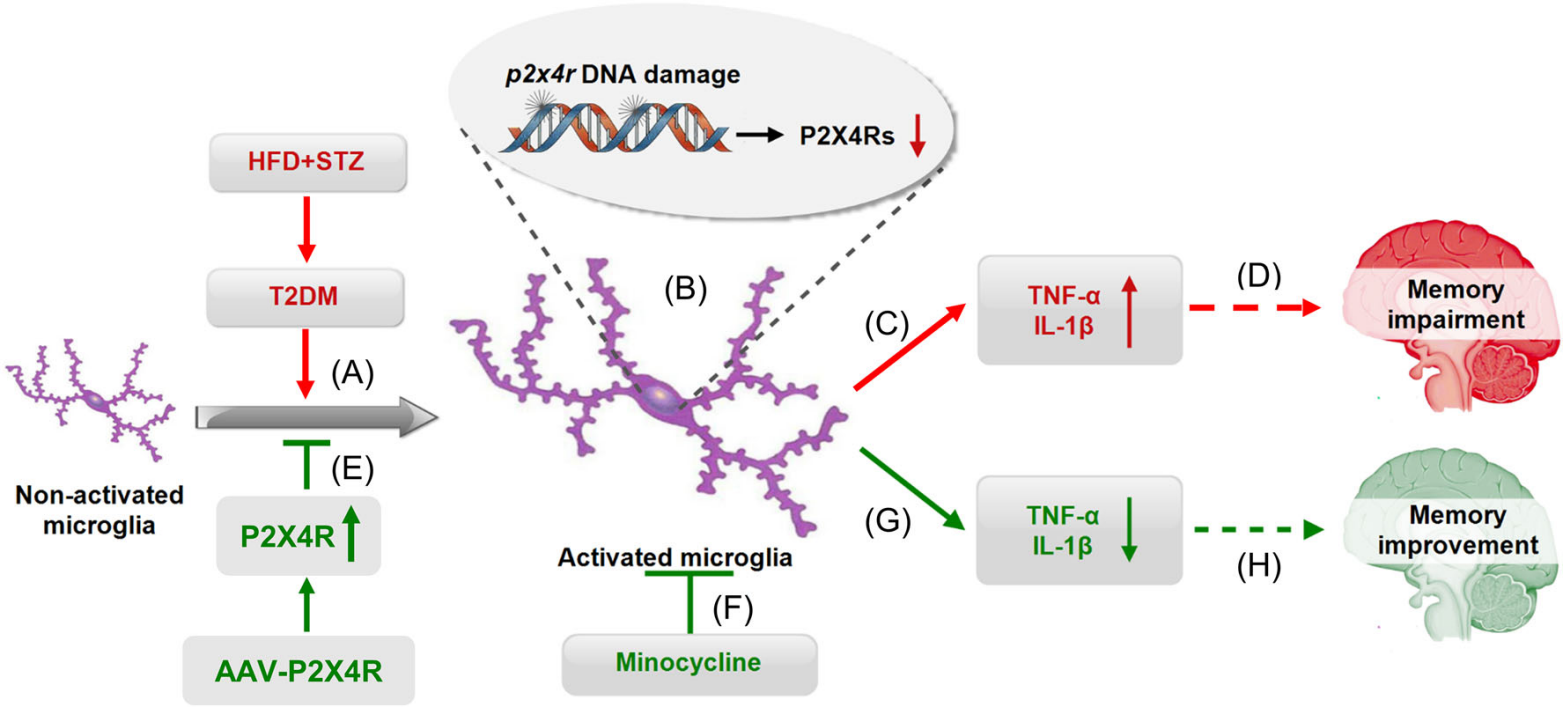
Working hypothesis. **A** T2DM induces excessive activation of microglia in the hippocampus, likely mediated by downregulated P2X4R expression. **B** DNA damage of the *p2x4r* gene leads to downregulated P2X4R expression. **C** Excessive activation of microglia results in enhanced release of TNF-α and IL-1β. **D** The released inflammatory mediators eventually lead to memory impairment in T2DM rats, **E**, **G**, **H** P2X4R overexpression counteracts the microglial activation and decreases the expression of TNF-α and IL-1β, eventually rescuing the memory impairment of T2DM rats. **F**, **G**, **H** Intervention by inhibiting microglial activation with minocycline successfully improves memory in T2DM rats.

Taken together, we conclude that: T2DM causes DNA damage to the *p2x4r* gene and the downregulation of P2X4R expression, resulting in the over-activation of microglia in the hippocampus, thus contributing to memory impairment in T2DM rats induced by an HFD and STZ injection. P2X4R over-expression or minocycline treatment improves memory in T2DM rats by blocking microglial activation (Fig. 10). These findings provide promising clues for the development of new therapeutic strategies for managing memory impairment in patients with type 2 diabetes.

## Electronic supplementary material

Below is the link to the electronic supplementary material.

Supplementary material 1 (PDF 276 kb)
