# Supplementary material for: Overexpression of Purinergic P2X4 Receptors in Hippocampus Rescues Memory Impairment in Rats with Type 2 Diabetes
Source: Neurosci Bull. 2020 Mar 20;36(7):719–32. doi: 10.1007/s12264-020-00478-7 (PMC7340685; doi:10.1007/s12264-020-00478-7)
Supplement: Supplementary file 1 — Supplementary material 1 (PDF 276 kb) [file 12264_2020_478_MOESM1_ESM.pdf]

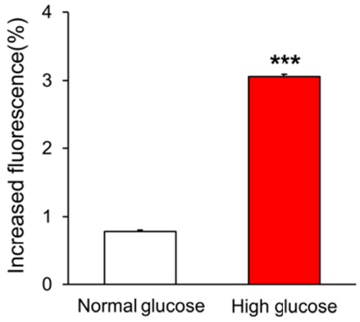

Fig. S1 High glucose induces reactive oxygen species (ROS) in cultured neurons. Neonatal hippocampal neurons were cultured with normal glucose (25 mmol/L) or high glucose (50 mmol/L) for 24 h. The high glucose significantly enhances ROS production as assessed by a ROS assay kit (Beyotime, Nantong, China).

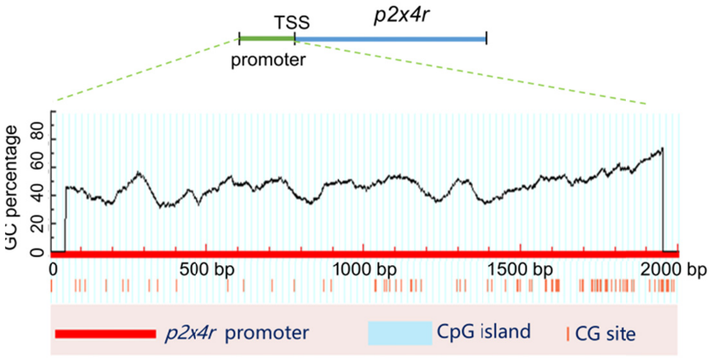

Fig. S2 Absence of CpG islands in the *p2x4r* gene promoter. The promoter sequence of *p2x4r* gene is 2000 bp before the transcriptional start site (TSS) in the NCBI Genbank database. There are no CpG islands in the *p2x4r* gene promoter according to online prediction by Methprimer software.
